# Assessment of metagenomic Nanopore and Illumina sequencing for recovering whole genome sequences of chikungunya and dengue viruses directly from clinical samples

**DOI:** 10.2807/1560-7917.ES.2018.23.50.1800228

**Published:** 2018-12-13

**Authors:** Liana E. Kafetzopoulou, Kyriakos Efthymiadis, Kuiama Lewandowski, Ant Crook, Dan Carter, Jane Osborne, Emma Aarons, Roger Hewson, Julian A. Hiscox, Miles W. Carroll, Richard Vipond, Steven T. Pullan

**Affiliations:** 1 Public Health England, National Infections Service, Porton Down, United Kingdom; 2 NIHR Health Protection Research Unit in Emerging and Zoonotic Infections, Liverpool, United Kingdom; 3 Artificial Intelligence Laboratory, Vrije Universiteit Brussel, Brussels, Belgium; 4 Rare and Imported Pathogens Laboratory, Public Health England, Porton Down, United Kingdom; 5 Institute of Infection and Global Health, University of Liverpool, United Kingdom

**Keywords:** chikungunya, dengue, metagenomic, nanopore, vector-borne infections, viral infections, dengue fever, chikungunya virus, dengue virus, surveillance, molecular methods, typing

## Abstract

**Background:**

The recent global emergence and re-emergence of arboviruses has caused significant human disease. Common vectors, symptoms and geographical distribution make differential diagnosis both important and challenging.

**Aim:**

To investigate the feasibility of metagenomic sequencing for recovering whole genome sequences of chikungunya and dengue viruses from clinical samples.

**Methods:**

We performed metagenomic sequencing using both the Illumina MiSeq and the portable Oxford Nanopore MinION on clinical samples which were real-time reverse transcription-PCR (qRT-PCR) positive for chikungunya (CHIKV) or dengue virus (DENV), two of the most important arboviruses. A total of 26 samples with a range of representative clinical Ct values were included in the study.

**Results:**

Direct metagenomic sequencing of nucleic acid extracts from serum or plasma without viral enrichment allowed for virus identification, subtype determination and elucidated complete or near-complete genomes adequate for phylogenetic analysis. One PCR-positive CHIKV sample was also found to be coinfected with DENV.

**Conclusions:**

This work demonstrates that metagenomic whole genome sequencing is feasible for the majority of CHIKV and DENV PCR-positive patient serum or plasma samples. Additionally, it explores the use of Nanopore metagenomic sequencing for DENV and CHIKV, which can likely be applied to other RNA viruses, highlighting the applicability of this approach to front-line public health and potential portable applications using the MinION.

## Introduction

Arboviruses are predominantly RNA viruses that replicate in haematophagous (blood-sucking) arthropod vectors such as ticks, mosquitoes and other biting flies to maintain their transmission cycle [1]. Human disease outbreaks caused by arboviruses have increased in prevalence since the 2000’s, led by the spread of mosquito-borne arboviruses such as chikungunya (CHIKV), dengue (DENV), West Nile (WNV), yellow fever (YFV) and Zika (ZIKV) viruses across both hemispheres [2]. CHIKV and DENV are of particular global health concern, as they have lost the need for enzootic amplification and consequently have caused extensive epidemics [3].

CHIKV is a single-stranded positive-sense RNA virus of the alphavirus genus, which causes the debilitating arthritic disease, chikungunya [4]. It has spread globally and been designated a serious emerging disease by the World Health Organization [5]. Outbreaks of CHIKV since 2005 have been associated with increased morbidity and possibly mortality [6,7].

DENV, which causes dengue, is a single-stranded positive-sense RNA virus of the flavivirus genus and the most prevalent human arboviral pathogen. Dengue occurs following infection with one of four DENV serotypes (DENV1–4). A minority of cases develop acute haemorrhagic manifestations and multi-organ failure. Despite DENV cases being under-reported, a 143.1% increased global incidence was estimated between 2005 and 2015 [8]. Approximately 500,000 DENV infected patients worldwide require hospitalisation annually [9].

Both CHIKV and DENV are predominantly transmitted to humans via *Aedes* species mosquitoes, particularly *Ae. aegypti* and *Ae. albopictus* [10,11], and share clinical presentations of arthralgia, headache, high fever, myalgia and rash. Circulation of CHIKV, DENV (and other arboviruses) in the same areas leads to challenges in differential diagnosis, especially in endemic regions in which diagnosis is predominantly symptom-based [12]. Additionally, reports of arboviral coinfections are increasingly common [13-16].

Metagenomic RNA sequencing allows for identification of multiple pathogens within a sample in a non-targeted and unbiased manner. It has identified causative agents in outbreaks, e.g. Lujo virus in South Africa [17], Bundibugyo ebolavirus in Uganda [18] and lead to novel virus discovery such as a rhabdovirus causing haemorrhagic fever in central Africa [19]. It also provides genomic information for typing and surveillance. Real-time genomic surveillance was facilitated on-site by the portable Oxford Nanopore MinION sequencer during the 2014–16 Ebola virus (EBOV) epidemic in West Africa and the 2015-16 ZIKV outbreak in the Americas [20-23] for epidemiological and transmission chain investigations [24]. In both examples, an amplicon sequencing approach was used, but viruses and bacteria from clinical, environmental and vector samples have been sequenced using metagenomic approaches on the MinION [25-28]. Metagenomic sequencing of CHIKV was demonstrated in principle on the MinION by Greninger et al. in 2015 reporting the detection of CHIKV from a human blood sample [28]. Additionally, Illumina-based metagenomics identified CHIKV coinfections within a ZIKV sample cohort [29], with the high proportion of CHIKV reads present making it a promising target for the approach.

In this study we set out to test the feasibility of direct metagenomic sequencing of DENV and CHIKV genomes from a cohort of clinical serum and plasma samples across a representative range of viral loads. The objective was to assess the proportion of viral nucleic acid relative to patient/background present in each sample and determine the sequencing limits for whole genome retrieval using both the laboratory-based Illumina technology and the portable MinION platform.

## Methods

### Sample collection and nucleic acid extraction

Twenty-six routine diagnostic samples, nine plasma and 17 serum, were obtained from the Rare and Imported Pathogens Laboratory (RIPL), Public Health England (PHE), Porton Down. All had previously tested positive by real-time reverse transcription-PCR (qRT-PCR) for chikungunya or dengue virus, with a maximum cut-off value of cycle threshold (Ct) 35. These samples had been selected based on their Ct values, among a larger set of 441 samples, so as to represent a Ct clinical range. Total nucleic acid was extracted from 140 μL of each using the QIAamp viral RNA kit (Qiagen, Hilden, Germany) replacing carrier RNA with linear polyacrylamide and eluting in 60 µL elution buffer provided in the kit, followed by treatment with TURBO DNase (Thermo Fisher Scientific, Waltham, United States (US)) at 37 °C for 30 min. RNA was purified and concentrated to 8 μL using the RNA Clean and Concentrator-5 kit (Zymo Research, Irvine, US).

### Molecular confirmation and quantification

Drosten et al. [30] and Edwards et al. [31] RT-PCR assays were used for confirmation of DENV and CHIKV respectively. RNA oligomers were used as standards for genome copy quantitation.

### Metagenomic cDNA reparation

Complementary DNA (cDNA) was prepared using a Sequence Independent Single Primer Amplification (SISPA) approach adapted from Greninger et al. [28]. Reverse transcription and second strand cDNA synthesis were as described [28]. cDNA amplification was performed using AccuTaq LA (Sigma, Poole, United Kingdom), in which 5μL of cDNA and 1 μL (100 pmol/μL) Primer B (5′-GTTTCCCACTGGAGGATA-3′) were added to a 50 μL reaction, according to manufacturer's instructions. PCR conditions were 98 °C for 30s, followed by 30 cycles of 94 °C for 15 s, 50 °C for 20 s, and 68 °C for 5 min, and a final step of 68 °C for 10 min. Amplified cDNA was purified using a 1:1 ratio of AMPure XP beads (Beckman Coulter, Brea, California (CA)) and quantified using the Qubit High Sensitivity dsDNA kit (Thermo Fisher, Waltham, US).

### MinION library preparation and sequencing

MinION sequencing libraries were prepared using total amplified cDNA of each sample to a maximum of 1 µg. Oxford Nanopore kits SQK-NSK007 or SQK-LSK208 (2D), SQK-LSK308 (1D^2^) and SQK-RBK001 (Rapid) were used and each sample was run individually on the appropriate flow cell (FLO-MIN105, FLO-MIN106 or FLO-MIN107) using the 48hr run script. Base calling was performed using Metrichor (ONT) for SQK-NSK007 and SQK-LSK208 or Albacore v1.2 for SQK-LSK308 and SQK-RBK001. Poretools [32] was used to extract FASTQ files from Metrichor FAST5 files.

### Illumina library preparation and sequencing

Nextera XT V2 kit (Illumina) sequencing libraries were prepared using 1.5 ng of amplified cDNA as per manufacturer's instructions. Samples were multiplexed in batches of a maximum of 16 samples per run and sequenced on a 2x150 bp-paired end Illumina MiSeq run, by Genomics Services Development Unit, PHE.

### Data handling

BWA MEM v0.7.15 [33] was used to align reads to the following references (GenBank ID): DENV Serotype 1 (NC_001477.1), DENV Serotype 2 (NC_001474.2), DENV Serotype 3 (NC_001475.2), DENV Serotype 4 (NC_002640.1) and CHIKV (NC_004162.2) using -x ont2d mode for Nanopore and MEM defaults for Illumina reads. Samtools v1.4 [34] was used to compute percentage reads mapped and coverage depth. Bedtools v2.26.0 [35] was used to calculate genome coverage at 10x and 20x. Mapping consensuses for Illumina were generated using in-house software QuasiBam [36] and for MinION using a simple pileup with bases called at a minimum depth of 20x and 70% support fraction. Nanopolish variants [24,37] was used in consensus mode to compute an error-corrected consensus sequence from the Rapid kit data. Taxonomic classification was performed using Kraken (0.10.4-beta) [38] and a locally built database populated with all RefSeq bacterial, viral, and archaeal genomes plus additional sequences [39]. De novo assemblies were generated using Spades 3.8.2 [40] in combination with SSPACE Standard v3.0 [41] for Illumina generated sequences and Canu v1.6 [41,42] for Nanopore sequences (settings: corOutCoverage = 1,000; genomeSize = 12,000; minReadLength = 300, minOverlapLength = 50). 

Consensus sequences for all samples tested are available in Genbank, raw fast5 files from 1D2 and 1D data (viral reads only) are deposited in SRA (Both under BioProject PRJNA508296).

## Results

### Metagenomic Illumina sequencing

**Figure 1:**
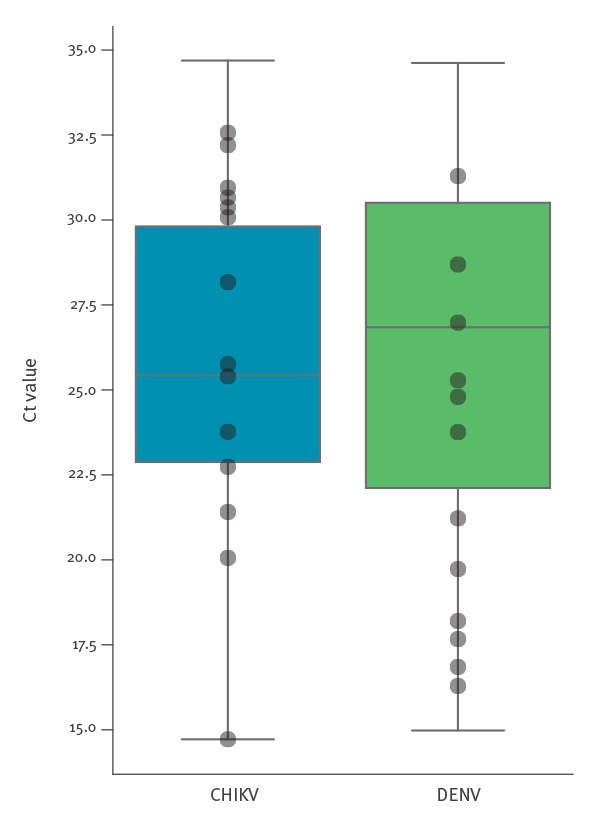
Cycle threshold (Ct) values distribution of chikungunya (n = 73) and dengue virus (n = 368) positive samples from the Rare and Imported Pathogens Laboratory, Public Health England, United Kingdom, 2016 (n = 441 total samples)

CHIKV: chikungunya virus; Ct: cycle threshold; DENV: dengue virus.

**Figure 2:**
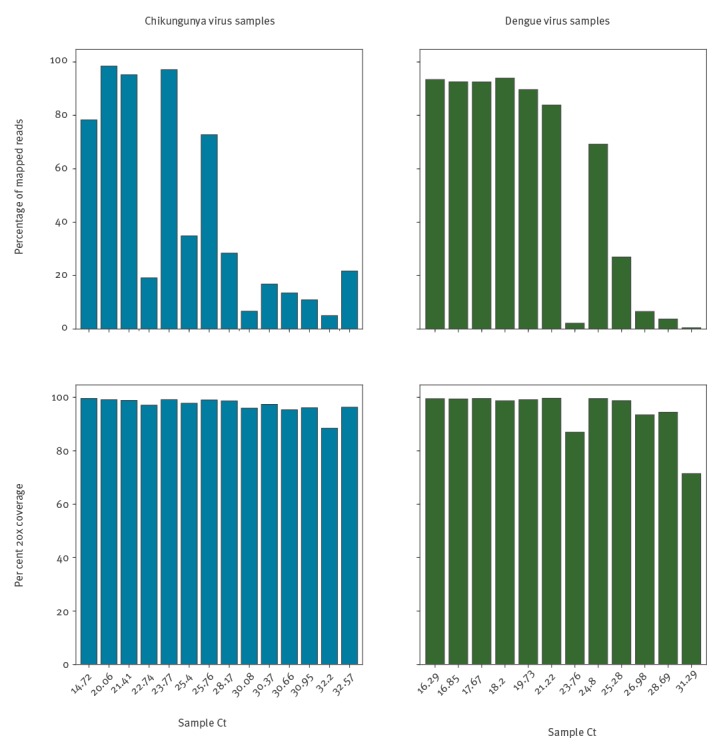
Proportion of reads mapping to the appropriate viral reference sequence and proportion of reference genome sequenced at minimum 20-fold coverage in each chikungunya or dengue virus positive sample, United Kingdom, 2017^a^ (n = 26 samples)

A total of 73 samples tested during 2016 in RIPL diagnostic laboratories, PHE Porton Down, were positive by qRT-PCR for CHIKV, and 368 were positive for DENV. Median Ct for CHIKV was 26.1, for DENV it was 26.8. For each virus, samples representing the range of viral titres seen during 2016 were selected, based on qRT-PCR Ct value (Figure 1). CHIKV samples selected (n = 14) ranged from Ct 14.72 to Ct 32.57, corresponding to 10^10^ and 10^5^ genome copies per mL of plasma or serum. DENV samples selected (n = 12) ranged from Ct 16.29 to Ct 31.29, corresponding to 10^9^ and 10^5^ genome copies per mL (Table 1). To measure the proportion of viral nucleic acid present relative to host/background and assess genome coverage, all samples were processed as described in methods and Illumina sequenced (Table 1). The proportion of total reads mapping to the respective viral reference was high for both viruses (Figure 2). In some low Ct samples, over 90% of reads mapped to the viral reference and proportions over 50% were still observed at mid-Ct range. The lowest proportions observed were 5.03% and 0.47% for CHIKV and DENV respectively (Table 1, Figure 2). The majority of samples returned over 95% genome coverage at 20x (21/26 samples) and over 98% genome coverage at 10x (20/26 samples). Irrespective of lower mapping percentages in high Ct value samples, genome coverage of 88.5% (20x) and 89.4% (10x) for CHIKV and 75.0% (20x) and 77.8% (10x) for DENV was observed.

The 14 CHIKV and 12 DENV samples selected for this work are indicated by circles. For each virus, the median Ct value of positive samples by quantitative real-time PCR is shown (horizontal line inside the box), as well as 25th and 75th percentiles (box lower and upper boundaries) and total range (whiskers).

**Table 1:** Description of samples positive for chikungunya and dengue virus by real-time reverse transcription-PCR with corresponding Illumina mapping data, United Kingdom, 2017^a^ (n = 26 samples)

Sample	Ct value	Estimated genome copy number in the sample (/mL)	Sample type	Total reads (R1 + R2)^b^	% reads mapping to reference viral genome	% 20x coverage	% 10x coverage	Reference virus^c^	Reference size (nts)
CHIKV 1	14.72	2.12E + 10	Plasma	1,113,560	78.32	99.59	99.72	CHIKV	11,826
CHIKV 2	20.06	5.49E + 08	Serum	1,278,624	98.48	99.14	99.47	CHIKV	11,826
CHIKV 3	21.41	2.18E + 08	Plasma	1,391,258	95.23	98.86	99.37	CHIKV	11,826
CHIKV 4	22.74	8.76E + 07	Plasma	888,968	19.16	97.08	97.32	CHIKV	11,826
CHIKV 5	23.77	4.33E + 07	Plasma	1,357,606	97.13	99.16	99.58	CHIKV	11,826
CHIKV 6	25.4	1.42E + 07	Serum	3,236,848	34.88	97.80	98.40	CHIKV	11,826
CHIKV 7	25.76	1.11E + 07	Plasma	3,748,070	72.77	99.04	99.56	CHIKV	11,826
CHIKV 8	28.17	2.13E + 06	Plasma	1,499,952	28.41	98.69	99.00	CHIKV	11,826
CHIKV 9	30.08	5.76E + 05	Serum	1,035,026	6.66	95.98	98.22	CHIKV	11,826
CHIKV 10	30.37	4.72E + 05	Serum	1,575,222	16.84	97.39	98.01	CHIKV	11,826
CHIKV 11	30.66	3.87E + 05	Serum	1,143,054	13.52	95.36	96.96	CHIKV	11,826
CHIKV 12	30.95	3.17E + 05	Serum	1,507,380	10.93	96.11	96.52	CHIKV	11,826
CHIKV 13	32.2	1.35E + 05	Serum	1,323,920	5.03	88.47	89.38	CHIKV	11,826
CHIKV 14	32.57	1.05E + 05	Serum	1,479,404	21.72	96.32	96.93	CHIKV	11,826
DENV 1	16.29	4.21E + 09	Plasma	439,292	93.44	99.51	99.58	DENV 1	10,735
DENV 2	16.85	2.83E + 09	Serum	513,472	92.56	99.40	99.58	DENV 1	10,735
DENV 3	17.67	1.58E + 09	Plasma	738,814	92.53	99.58	99.58	DENV 2	10,723
DENV 4	18.20	1.09E + 09	Serum	477,368	93.97	98.73	99.12	DENV 2	10,723
DENV 5	19.73	3.67E + 08	Serum	915554	89.65	99.14	99.40	DENV 2	10,723
DENV 6	21.22	3.61E + 07	Serum	3,587,926	83.87	99.68	99.69	DENV 4	10,649
DENV 7	23.76	2.11E + 07	Serum	4,146,678	2.17	86.99	89.13	DENV 1	10,735
DENV 8	24.8	1.01E + 07	Serum	777,264	69.23	99.56	99.58	DENV 3	10,707
DENV 9	25.28	7.17E + 06	Plasma	787,728	26.97	98.77	98.81	DENV 2	10,723
DENV 10	26.98	2.15E + 06	Serum	596,240	6.58	93.47	93.97	DENV 3	10,707
DENV 11	28.69	6.39E + 05	Serum	1,034,698	3.73	94.44	94.70	DENV 1	10,735
DENV 12	31.29	1.01E + 05	Serum	1,374,766	0.47	71.46	77.76	DENV 1	10,735

CHIKV: chikungunya virus; Ct: cycle threshold; DENV: dengue virus.

^a ^The Illumina mapping data presented in the table were obtained in 2017 on samples that had been collected and found positive for chikungunya or dengue virus by real-time reverse transcription-PCR in 2016.

^b^ ‘R1 + R2’ indicates paired-end sequencing.

^c^ For DENV the serotype is also indicated.

Ct: cycle threshold.

^a ^The Illumina data presented in the figure were obtained in 2017 on samples that had been collected and found positive for chikungunya or dengue virus by real-time reverse transcription-PCR in 2016.

The percentage of total reads mapping to the appropriate reference sequence is plotted in the upper panel. Lower panels display the percentage of the reference genome sequenced to a minimum depth of 20-fold in the Illumina data.

### Metagenomic MinION sequencing

Four representative samples for each virus were selected for Nanopore sequencing (Table 2).

**Table 2:** Description of chikungunya and dengue virus positive samples by real-time reverse transcription-PCR and corresponding Nanopore sequencing data, United Kingdom, 2017^a^ (n = 8 samples)

Sample	Ct value	cDNA amount used for the library (ng)	Sequencing kit (2D kit version)	Flow cell (FLO-)	1D total bp	1D total reads	1D mean read length (nt)	1D max read length (nt)
CHIKV 1	14.7	431.5	SQK-NSK007	MIN105	1.51E + 08	267,171	564	92,712
CHIKV 3	21.4	928.8	SQK-LSK208	MIN106	1.63E + 09	1,891,028	862	99,031
CHIKV 4	22.7	113.4	SQK-NSK007	MIN105	1.74E + 08	216,493	805	125,387
CHIKV 9	30.1	212.4	SQK-LSK208	MIN106	2.12E + 09	3,481,358	608	121,711
DENV 1	16.3	1,626.0	SQK-NSK007	MIN105	2.42E + 08	284,622	851	115,494
DENV 2	16.9	1,626.0	SQK-NSK007	MIN105	1.55E + 08	203,700	760	52,157
DENV 6	21.2	475.0	SQK-LSK208	MIN106	1.22E + 09	1,377,721	886	118,733
DENV 11	28.7	65.8	SQK-LSK208	MIN106	7.07E + 08	1,111,566	636	119,438

Ct: cycle threshold.

^a^ The Nanopore data presented in the table were obtained in 2017 on samples that had been collected and found positive for chikungunya or dengue virus by real-time reverse transcription-PCR in 2016.

**Figure 3:**
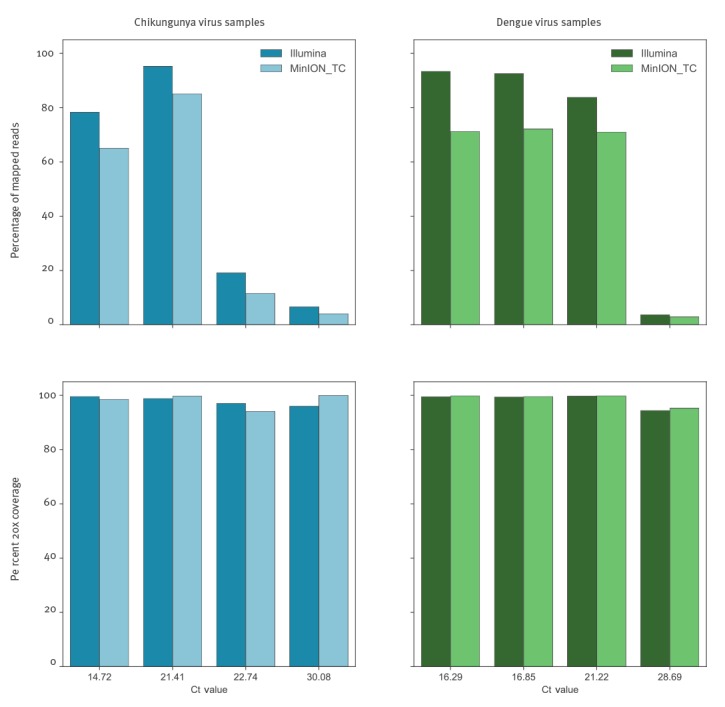
Comparison of Nanopore and Illumina results, as to proportions of reads mapping to the appropriate reference viral sequence, and proportions of reference genome sequenced at minimum 20-fold coverage, United Kingdom, 2017^a^ (n = 8 samples)

Figure 3 shows percentages of reads mapping to viral reference, which were generally concordant with the Illumina data, although a slight decrease is observed across the range of Ct values. In the Nanopore data, the highest mapped read percentages observed were 85.12% and 72.14% for CHIKV3 and DENV 2 respectively, compared with 95.23% and 92.56% in the Illumina data from the same samples. While in high Ct samples the viral proportion drops to 4.08% for CHIKV 9 and 2.90% for DENV 11, from 6.66% and 3.73% in the Illumina data. 

Ct: cycle threshold; TC: template/complement.

^a^ The Nanopore and Illumina data presented in the figure were obtained in 2017 on samples that had been collected and found positive for chikungunya or dengue virus by real-time reverse transcription-PCR in 2016.

The percentage of total reads mapping to the appropriate reference sequence is plotted in the upper panel. Lower panels display the percentage of the reference genome sequenced to a minimum depth of 20-fold in the data generated, in dark blue or dark green for the Illumina sequence data, in light blue or light green for Nanopore data (MinION_TC).

Despite the decrease in proportion of mapped viral reads, comparable genome coverage is observed at both 20x and 10x (Figure 3, Table 3) and is even increased compared with Illumina data at lower viral titres, e.g. 100% at 20x for CHIKV 9 compared with 95.98% in the Illumina data and 95.25% for the high Ct DENV 11 sample, which generated 94.44% coverage from the Illumina data. Average read lengths in Nanopore data ranged from 564 to 886 bp (Table 2).

**Table 3:** Summary of Nanopore mapping data on chikungunya and dengue virus positive samples by real-time reverse transcription-PCR, United Kingdom, 2017^a^ (n = 8 samples)

Sample	Ct value	Total reads	% reads mapping to appropriate viral sequence	% 20x coverage	20x genome length (nt)	% 10x coverage	Reference^b^	Reference size (nt)	Max de novo contig (nt)
CHIKV 1	14.7	267,171	65.1	98.57	11,658	99.2	CHIKV	11,826	5,263
CHIKV 3	21.4	1,891,028	85.1	99.76	11,798	99.9	CHIKV	11,826	10,793
CHIKV 4	22.7	216,493	11.6	94.11	11,130	97.2	CHIKV	11,826	4,256
CHIKV 9	30.08	3,481,358	4.08	100	11,826	100	CHIKV	11,826	9,860
DENV 1	16.3	284,622	71.3	99.9	10,719	99.9	DENV 1	10,735	8,281
DENV 2	16.9	203,700	72.1	99.6	10,692	99.6	DENV 1	10,735	10,157
DENV 6	21.2	1,377,721	71.1	99.9	10,634	99.9	DENV 4	10,649	7,877
DENV 11	28.7	1,111,566	2.9	95.3	10,226	96.3	DENV 1	10,735	4,699

CHIKV: chikungunya virus; Ct: cycle threshold; DENV: dengue virus.

^a^ The Nanopore data presented in the table were obtained in 2017 on samples that had been collected and found positive for chikungunya or dengue virus by real-time reverse transcription-PCR in 2016.

^b^ For DENV the serotype is also indicated.

**Figure 4:**
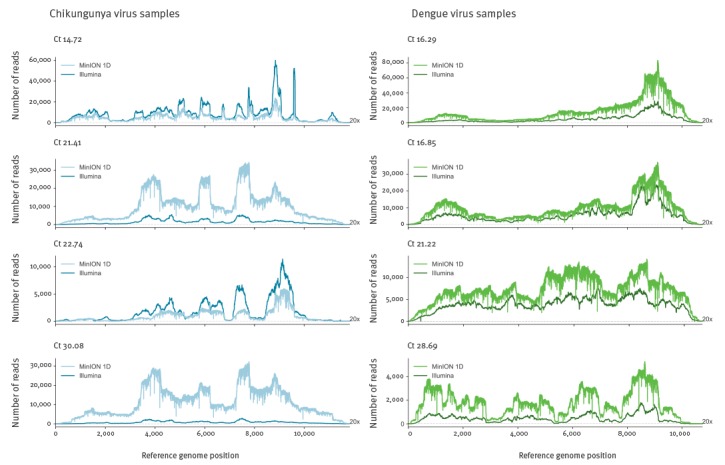
Coverage depth across the chikungunya or dengue viral genome, United Kingdom, 2017^a^ (n = 8 samples)

Figure 4 shows coverage depth of reads mapped across the relevant genome for each sample sequenced by both Illumina and Nanopore. Read levels are not normalised thus actual depth is a function of total reads sequenced, but the pattern of coverage seen is highly similar suggesting it is more dependent upon the SISPA methodology than sequencing library preparation. From Nanopore consensus genome sequences, between 99.93% and 100% of bases called per sample agreed with the Illumina generated sequence.

Ct: cycle threshold.

^a^ The Nanopore and Illumina data presented in the figure were obtained in 2017 on samples that had been collected and found positive for chikungunya or dengue virus by real-time reverse transcription-PCR in 2016.

Each graph corresponds to a given sample, defined by its Ct value. Read depth (y-axis) across the genome (x-axis) following reference alignment is shown. Illumina coverage is shown in darker blue and darker green for chikugunya and dengue virus positive samples respectively. Nanopore (MinION) coverage is indicated in lighter blue or lighter green for chikungunya and dengue virus positive samples respectively. Total depth has not been normalised; comparison is to show overall pattern of coverage is highly similar across the methods. Dotted horizontal line indicates depth of 20x coverage, used for consensus calling.

### Metagenomic data analysis and coinfection identification

**Figure 5:**
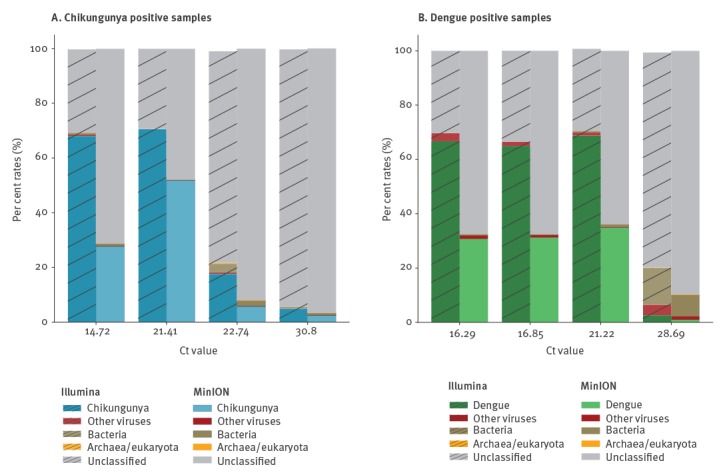
Kraken classification of reads from metagenomic sequencing in (A) chikungunya and (B) dengue real-time reverse transcription-PCR positive samples, United Kingdom, 2017^a^ (n = 8 samples)

To test the applicability of a metagenomic analysis approach to the data, we assessed read taxonomic classification using Kraken (Figure 5). The distribution of reads classified as CHIKV, DENV, other viruses, bacteria, and archaea/eukaryota show a similar pattern for Illumina and Nanopore data. The proportion of unclassified reads for each sample increased with Ct value, as the proportion of human origin reads is higher and the human genome is not represented in our Kraken database. A decrease in the percentage of CHIKV and DENV classified reads is observed for MinION data compared with Illumina, but was sufficient to identify the correct predominant virus in all samples.

Ct: cycle threshold.

^a^ Read taxonomic classification using Kraken was conducted in 2017 on samples that had been collected and found positive for chikungunya or dengue virus by real-time reverse transcription-PCR in 2016.

Kraken classification distribution comparison for Illumina (cross-hatched) and Nanopore data. Reads grouped as either chikungunya virus (blue in panel A), dengue virus (green in panel B), other viruses (brown), archea/eukaryota (orange), bacteria (brown) or unclassified (grey).

Kraken analysis also allowed for the identification of a DENV coinfection in sample CHIKV 3, the consensus sequence of which was unique in the sample set, eliminating cross-contamination from the DENV positive samples as potential source. Kraken classified 0.08% of Illumina reads and 0.15% of MinION reads as DENV. Using reference mapping to validate the finding, 0.22% of Illumina reads and 0.43% of MinION reads mapped to a DENV-1 reference genome. Genome coverage at 20x of 99.73% and 95.99% was achieved for the primary CHIKV and secondary DENV coinfection respectively, with a single MinION flow cell.

### De novo assembly

De novo assembly of the data was attempted using Canu [42] and contigs identified using Basic Local Alignment Search Tool against a Nt database (BLASTn). Table 3 lists the longest viral contig length identified in each sample, ranging from 4.2 Kb (36% of reference genome size) to 10.8 Kb (91%) for CHIKV and 4.7 Kb (44%) to 10.1 Kb (95%) for DENV. Identification of the pathogen present without prior knowledge would have therefore been possible for all samples.

### Updated MinION library kits

**Figure 6:**
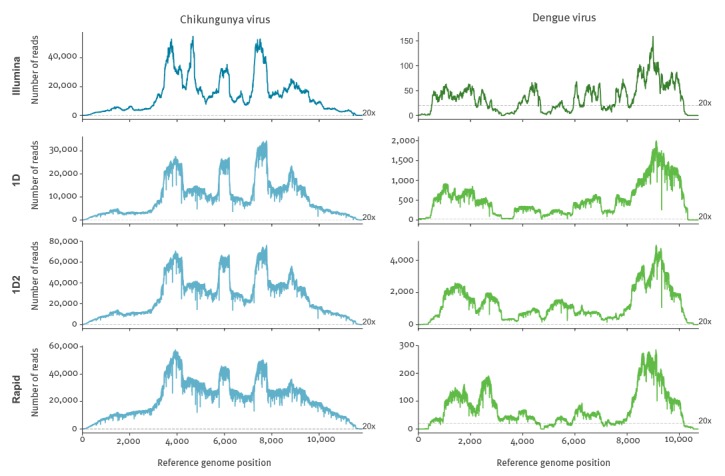
Comparison of genome coverage depth across the chikungunya virus or dengue virus genome for different sequencing library preparation methods in a sample coinfected with dengue and chikungunya viruses, United Kingdom, 2017^a^ (n = 1 sample)

^a ^Results presented in the Figure were obtained in 2017 on a sample that had been collected and found positive for chikungunya virus by real-time reverse transcription-PCR in 2016. In 2017, the sample was further found to be coinfected with CHIKV and DENV by metagenomic sequencing.

We repeated the sequencing of the coinfected CHIKV 3 sample using the MinION 1D^2^ (SQK-LSK308) and Rapid (SQK-RBK001) kits, currently the most accurate and the fastest library preparation kits available, respectively. Using the 1D^2^ kit 74.5% of reads generated mapped to CHIKV and 0.37% to DENV, while from the Rapid kit the result was 66.26% and 0.29% respectively (both lower than observed in the 2D chemistry). Coverage at 20x for CHIKV was above 99% for both kits, and for DENV was 95.04% from the 1D^2^ and 81.09% from the Rapid kit (Table 4). Coverage depth pattern across the genome for both viruses (Figure 6) was similar for all library kits tested. Near-maximum coverage for both viruses was obtained within 30 min with the 2D kit, 8 min with the 1D^2^ kit and 85 min with the Rapid kit (Supplementary Figure 1). De novo assembly (Table 4) produced best CHIKV contigs of 10.7, 11.3 and 11.4 Kb for the 2D, 1D^2^ and Rapid libraries respectively and the longest contigs generated for DENV were 7.5, 2.2 and 4.2 Kb.

**Table 4:** Comparison of Nanopore mapping data across library kits, United Kingdom, 2017^a^ (n = 8 samples)

Platform	Kit information	Flow cell (FLO-)	Virus identified	Total reads (nt)	% reads mapping	% 20x coverage	% 10x coverage	Reference^b^	Reference size (nt)	Max de novo contig (nt)
Illumina	Nextera XT	NA	CHIKV	1,391,258	95.23	98.86	99.37	CHIKV	11,826	7,321
Illumina	Nextera XT	NA	DENV	1,391,258	0.22	63.66	77.82	DENV1	10,735	6,613
MinION 2D	SQK-LSK208	MIN106	CHIKV	1,891,028	85.12	99.73	99.91	CHIKV	11,826	10,793
MinION 2D	SQK-LSK208	MIN106	DENV	1,891,028	0.43	95.99	96.09	DENV1	10,735	7,549
MinION 1D^2^	SQK-LSK308	MIN107	CHIKV	5,080,906	74.50	99.94	100	CHIKV	11,826	11,369
MinION 1D^2^	SQK-LSK308	MIN107	DENV	5,080,906	0.37	95.04	96.42	DENV1	10,735	2,199
MinION Rapid	SQK-RBK001	MIN106	CHIKV	611,110	66.26	99.66	99.68	CHIKV	11,826	11,473
MinION Rapid	SQK-RBK001	MIN106	DENV	611,110	0.29	81.09	90.83	DENV1	10,735	4,227

CHIKV: chikungunya virus; Ct: cycle threshold; DENV: dengue virus. NA: not applicable.

^a ^Results presented in the table were obtained in 2017 on samples that had been collected and found positive for chikungunya or dengue virus by real-time reverse transcription-PCR in 2016.

^b^ For DENV the serotype is also indicated.

Read depth across both CHIKV and DENV genomes following reference alignment is shown for coinfection sample CHIKV 3, sequenced using four different sequencing library preparation/sequencing methods. Total coverage depth has not been normalised; comparison is to show overall pattern of coverage is highly similar across the methods. Dotted horizontal line indicates depth of 20x coverage, used for consensus calling.

The 1D data from the Rapid kit was sufficient to call a consensus from 11,647/11,826 bases of the CHIKV reference with 179/11,826 bases called as ambiguous or too low coverage. All bases called were concordant with the Illumina consensus. A polishing step using Nanopolish [37] with a subset of the mapped reads (ca 100x coverage depth) significantly reduced ambiguous calls to 90/11,826, introducing a single disagreement with the Illumina consensus (99.99% concordance). Despite considerably greater read depth, the 1D^2^ kit called only 11,082/11,826 due to a higher proportion, 744/11826, of ambiguous base calls, suggesting 1D reads are most suitable for this approach.

## Discussion

These results clearly show that there are considerable levels of viral nucleic acid present in a large proportion of CHIKV and DENV qRT-PCR positive clinical samples, and demonstrate that relatively modest metagenomic sequencing is capable of elucidating significant portions of viral genome even for samples with Ct values at the higher end of clinical range. A decreased Ct value coincided with an increased proportion of viral reads, with a considerable level of variation between samples, likely because of the total level of non-viral host/background nucleic acid present due to variability between patients or in sample handling during collection, storage and testing. For example, the two lowest viral titre CHIKV samples (13 and 14) have similar Ct values (32.2 and 32.57) but varied significantly in the proportion of viral reads (5.03% and 21.72%). The 5.03% viral reads in CHIKV13 is the lowest for CHIKV, yet still sufficient to generate 88.5% of the CHIKV genome at 20x depth from just ca 662,000 paired-end Illumina reads. This amount of genomic information is highly informative and further sequencing would likely increase coverage. Only seven of the 73 total CHIKV diagnostic samples tested in 2016 had a Ct greater than 32.2 (including sample CHIKV14) (Table 1), which suggests that for the majority (> 90%) of CHIKV PCR positive samples, viral load is sufficient for genome sequencing directly from patient samples without further viral enrichment beyond a simple DNAse digestion (Figure 1). The lowest viral read proportion observed in the DENV samples was 0.47% in DENV12, Ct 31.29, which generated 71.5% coverage at 20x depth (increased to 77.8 at 10x depth) from just 687,000 paired end Illumina reads and allowed for DENV serotype identification. Only 62 of 368 DENV cases in 2016 had a higher Ct, predicting that > 80% of PCR positive DENV samples have a viral load sufficient for genome sequencing (Figure 1). These estimates are based on Ct range distribution from a single year, results may vary from year to year. 

The high yield of viral sequences from clinical CHIKV and DENV samples make the exciting prospect of metagenomic MinION viral whole-genome-sequencing feasible, even for lower viral titre samples. Evaluating this on a representative subset of our samples demonstrates that viral read proportions are in general agreement with that seen for Illumina sequencing, predicting a similar proportion of qRT-PCR positive patient samples would be suitable for direct metagenomic sequencing on the MinION. Differences in precise proportions of viral reads seen between Illumina and MinION are likely due to inter-library variation. Differences in genome coverage achieved are due to both differences in total reads generated per sample (not normalised between platforms) as well as differences in average read length. Of the samples tested on the MinION, the lowest titre samples CHIKV 9 and DENV 11 both generated near complete genome coverage. 

We repeated the sequencing of the coinfected CHIKV 3 sample using the MinION 1D^2^ (SQK-LSK308) and Rapid (SQK-RBK001) kits. A reduction in viral proportion of total reads was observed compared with the 2D kit, which may be due partly to the extended storage time of the original samples before retesting. In the case of the 1D^2^ kit, the lower proportion was outweighed by a substantial increase in total data generated per flow cell (5 M vs 1.8 M reads). For the Rapid kit, the total data produced should be considered in the light of the greatly simplified sample workflow and turnaround-time.

The use of metagenomics to elucidate genomic sequences of RNA viruses directly from clinical samples has several obvious benefits in public health applications. The primary benefit over targeted methods is the hypothesis-free nature of the assay, which allows identification and genomic characterisation of novel or unexpected RNA viral agents, either as primary or coinfectants (demonstrated here in the CHIKV/DENV coinfection sample), without any prior clinical knowledge. It also removes the need for laboratory optimisation of targeted methods, such as primer or bait-probe design and testing, and is not subject to escape mutations in target sites that afflict targeted sequencing and diagnostic methods. This issue particularly relevant for highly diverse RNA viruses, such as Lassa virus, which are difficult to assess using targeted methods, without regular reappraisal [43].

The principal limitation of the metagenomic approach is the limit of detection. The data generated here show that viral titres as low as 10^5^ are sufficient for significant genome recovery by this method, but ZIKV is a recent example of a pathogen typically present at lower clinical titres, for which targeted methods are an absolute requirement [22,23]. For diagnostic purposes qRT-PCR has a lower limit of detection, provided the target site is conserved in the pathogen isolate tested. Clearly no single method is most suitable for both detection and genotyping of all pathogens and each has a role to play in differing circumstances.

The ability to generate genomic data directly from patient samples is clearly of great benefit to public health (reviewed in detail [44]). It can be used in a routine surveillance capacity or early during suspected outbreaks to link related cases who may be missed by traditional epidemiology [45] and identify outbreak cases distinct from typically circulating seasonal strains, which is key in regions endemic for the pathogen in question. The use of whole genome sequences offers the greatest precision for these applications, compared with typing methods based on specific genomic regions [44]. Whole genome sequencing on a portable device allows this information to be generated rapidly and within the affected region [24], enabling timely identification of an outbreak, or allaying fears of a potential one if cases are not linked. Furthermore mutations relating to viral drug resistance or pathogenicity can be monitored [44]. Therefore the ability to generate near-complete viral genome sequences directly from clinical samples on a portable sequencing device has many potential applications.

## Conclusions

We demonstrate that across the clinically relevant range of viral loads an unexpectedly high proportion of reads generated metagenomically from CHIKV and DENV clinical samples are viral in origin. Therefore metagenomic sequencing provides an effective approach for the analysis of CHIKV and DENV genomes directly from the majority of qRT-PCR positive serum and plasma samples, without the need for culture or viral nucleic acid enrichment beyond a simple DNA digestion. We demonstrate this is equally possible on the Oxford Nanopore MinION, making metagenomic whole genome sequencing potentially feasible in the field.
